# Associations between daily ambient temperature and sedentary time among children 4–6 years old in Mexico City

**DOI:** 10.1371/journal.pone.0241446

**Published:** 2020-10-30

**Authors:** Sandy Wong, Alejandra Cantoral, Martha María Téllez-Rojo, Ivan Pantic, Emily Oken, Katherine Svensson, Michael Dorman, Iván Gutiérrez-Avila, Johnathan Rush, Nia McRae, Robert O. Wright, Andrea A. Baccarelli, Itai Kloog, Allan C. Just

**Affiliations:** 1 Department of Environmental Medicine & Public Health, Icahn School of Medicine at Mount Sinai, New York, New York, United States of America; 2 Centro de Investigacion en Nutrición y Salud, Instituto Nacional de Salud Pública, Cuernavaca, Morelos, Mexico; 3 Department of Developmental Neurobiology, National Institute of Perinatology, Mexico City, Mexico; 4 Division of Chronic Disease Research Across the Lifecourse, Department of Population Medicine, Harvard Medical School and Harvard Pilgrim Health Care Institute, Boston, Massachusetts, United States of America; 5 Department of Geography and Environmental Development, Ben-Gurion University of the Negev, Beer-Sheva, Israel; 6 Columbia University Mailman School of Public Health, New York, New York, United States of America; University of New Mexico, UNITED STATES

## Abstract

**Background:**

Sedentary behavior is a worldwide public health concern. There is consistent and growing evidence linking sedentary behavior to mortality and morbidity. Early monitoring and assessment of environmental factors associated with sedentary behaviors at a young age are important initial steps for understanding children’s sedentary time and identifying pertinent interventions.

**Objective:**

This study examines the association between daily temperature (maximum, mean, minimum, and diurnal variation) and all-day sedentary time among 4–6 year old children in Mexico City (n = 559) from the year 2013 to 2015.

**Methods:**

We developed a spatiotemporally resolved hybrid satellite-based land use regression temperature model and calculated percent daily sedentary time from aggregating 10-second epoch vertical counts captured by accelerometers that participants wore for one week. We modeled generalized additive models (GAMs), one for each temperature type as a covariate (maximum, mean, minimum, and diurnal variation). All GAMs included percent all-day sedentary time as the outcome and participant-level random intercepts to account for repeated measures of sedentary time. Our models were adjusted for demographic factors and environmental exposures.

**Results:**

Daily maximum temperature, mean temperature, and diurnal variation have significant negative linear relationships with all-day sedentary time (p<0.01). There is no significant association between daily minimum temperature and all-day sedentary time. Children have on average 0.26% less daily sedentary time (approximately 2.2 minutes) for each 1°C increase in ambient maximum temperature (range 7.1–30.2°C), 0.27% less daily sedentary time (approximately 2.3 minutes) for each 1°C increase in ambient mean temperature (range 4.3–22.2°C), and 0.23% less daily sedentary time (approximately 2.0 minutes) for each 1°C increase in diurnal variation (range 3.0–21.6°C).

**Conclusions:**

These results are contrary to our hypothesis in which we expected a curvilinear relationship between temperature (maximum, mean, minimum, and diurnal variation) and sedentary time. Our findings suggest that temperature is an important environmental factor that influences children’s sedentary behavior.

## Introduction

Sedentary behavior is a worldwide public health concern as there is consistent and growing evidence linking sedentary behavior to mortality and numerous morbidities [1]. Children are a particularly critical age group for assessing and tracking sedentary behavior over time [2–4]. For children, sedentary behavior is associated with poorer physical and mental health outcomes [3, 4], including obesity, high blood pressure and total cholesterol, lower physical fitness, social behavior problems, lower self-esteem, poor cognitive development, and lower academic performance [1]. Furthermore, both human and animal studies find that active children are more likely to be active later in life as adults [5, 6]. These findings suggest that physical activity behaviors developed in childhood remain stable over time, and that early monitoring and intervention of sedentary behaviors in childhood are important for positive changes over the life course.

To date, researchers have investigated numerous factors associated with physical activity and sedentary behavior. In a systematic review conducted by Van Der Horst and colleagues, they evaluated studies published from 1999–2005 for correlates of physical activity and sedentary behavior among children, and broadly categorized relevant correlates as environmental, demographic, biological, psychological, and sociocultural factors [7]. They identified a gap in the literature on physical environmental correlates and a need for future research to utilize objective measurements of the environment as most existing studies relied on self-reported data [7]. Since the time of their review, new studies on physical activity and sedentary behavior have analyzed various environmental attributes, including greenness, parks and playgrounds, noise, season, precipitation, daylight, humidity, and temperature [8–12].

Seasonality is now recognized as an important influence on children’s health behaviors [13], with increasing evidence to support the effects of seasonal variation on children’s physical activity–as well as sedentary behavior but to a lesser extent. Studies largely find that children’s levels of moderate-to-vigorous physical activity (MVPA) are significantly lower in the winter compared to other seasons in different contexts, including Australia [14], Canada [15], Norway [16], and the UK [13]. However, there are inconsistencies as to which seasons children have significantly different MVPA relative to winter. For example, children have lower MVPA in winter compared to autumn in Canada [15], spring and summer in Australia [14], and summer and autumn in Norway [16]. In the UK, children have lower MVPA in autumn and winter relative to spring [13]. As for sedentary behavior, studies find an increase in sedentary time in winter and autumn relative to spring in the UK [13], and spring and summer in Norway [16].

There are acknowledged limitations to using seasonality [17, 18]. Season is a proxy for average weather variations for four distinct time periods of the year (i.e., spring, summer, autumn, winter) or less in more temperate climates, thus obscuring day-to-day temporal variations in weather conditions such as sudden spikes and drops in temperature. Moreover, one season is presumed to have considerably different weather patterns from other seasons, an assumption that is unlikely for the days and weeks immediately before and after a transition from one season to another. Definitions of seasons also vary across different regions of the world, restricting international comparisons of findings on seasonal effects on levels of physical activity [17, 18].

Daily weather conditions have been used in lieu of seasonality in physical activity studies, of which temperature is always included as a correlate. Furthermore, there is considerable intraindividual variability in children’s physical activity levels [19] and researchers have correspondingly analyzed intraindividual associations between daily weather conditions and children’s physical activity [17, 18, 20]. Findings on the relationship between temperature and physical activity are varied, with both linear and non-linear associations that are influenced by geography [21–24]. Studies in Montreal and Auckland observe a linear relationship, with higher temperatures corresponding to higher levels of physical activity [25–27]. Research in Australia identify a curvilinear fit between temperature and children’s MVPA whereby levels of MVPA peaked from 20–22°C in one study [17] and 20–25°C in another study [18], followed by a decline in MVPA at higher temperatures [17, 18]. In another study using data from 17 studies across the world, researchers observe an increase in temperature alongside an increase in physical activity from 0–20°C, followed by a decrease in physical activity at temperatures higher than 20°C [20]. Across these studies, limitations include using weather data from a single ground station to represent the entire study location rather than participants’ home location and analyzing data in high income countries [20].

Compared to physical activity, much less research has investigated the role of temperature on children’s sedentary behavior. Among the limited research on temperature and sedentary time, there are inconclusive findings and different data collection methods. One study based in the US found that levels of sedentary behavior declined at the extreme ends of mean temperature (<18.3°C and ≥18.3°C) [28]. Researchers used temperature data from one weather station to represent a central location for the entire study area [28]. Another study found no association between maximum temperature and sedentary time among European children and utilized data from the weather station closest to participants’ school location [29].

The vast majority of studies on temperature and physical activity or sedentary behavior have taken place in the US, Canada, Western Europe, and Australia [21–24, 28, 29], with rare exceptions based in non-Western regions. It remains important to investigate the relationship between temperature and sedentary behavior in a wider range of climates and countries in order to assess how variations in weather conditions, social norms, and institutional and infrastructural resources are linked to differences in activity behaviors [16, 24].

Our study seeks to contribute to the aforementioned research gaps by investigating the relationship between daily temperature and all-day sedentary time among children in Mexico. In general, there is a need for more research analyzing the connections between temperature and children’s health behaviors and outcomes–and none have focused on Mexico, a middle-income country. The majority of investigations on temperature and health either study the general population or focus on health conditions in high-income countries that are specific to adults or the elderly [30, 31]. Additionally, we use weather data based on participants’ home location.

We hypothesized the relationship between temperature (maximum, mean, minimum, and diurnal variation) and sedentary time to be curvilinear. Existing research has found that higher temperatures at the extremes (i.e., too hot or too cold) are associated with reduced physical activity [21, 23, 28]. These earlier findings suggest that sedentary behavior increases when temperatures are too high or too low.

## Methods and materials

### Environmental exposure variables

#### Temperature

Our temperature estimates were generated from a spatiotemporally resolved hybrid satellite-based land use regression temperature model that we developed for the Megalopolis of Central Mexico, which includes Mexico City and surrounding metropolitan areas. Our model predicted daily ambient temperature on a 1x1 km grid using data from two National Aeronautics and Space Administration (NASA) satellites (Aqua and Terra) and 153 meteorological ground stations. We used mixed-effects regression models with daily random effects for each year (2013, 2014, and 2015) and temperature type (maximum, mean, and minimum). Predictors included satellite-based land surface temperature (plus temporal imputation and indicators for missingness), monthly Normalized Difference Vegetation Index (NDVI), elevation, wind speed and flexible terms for seasonality. Model performance was assessed with group ten-fold cross validation at withheld monitors and root mean squared error ranges (and R^2^ ranges) for the three years were 1.69 to 1.76 (R^2^: 0.85–0.88) for maximum temperature, 1.07 to 1.22 (R^2^: 0.90–0.93) for mean temperature, and 1.62 to 1.78 (R^2^: 0.81–0.85) for minimum temperature. Our data and R code are available in an open research repository [32].

#### NDVI

NDVI is a common indicator of green vegetation and ranges in value from -1 to 1. A higher NDVI denotes a higher density of green vegetation. The average NDVI value was calculated for each season (cold-dry from November to February, warm-dry from March to April, and rainy from May to October) using 30 x 30-meter pixel Landsat 5 Collection 1 Tier 1 TOA Reflectance, Landsat 7 Collection 1 Tier 1 TOA Reflectance, and Landsat 8 Collection 1 Tier 1 TOA Reflectance. A buffer of 250 meters was created around each participant’s place of residence and the average NDVI was calculated within each buffer. We also considered the NDVI of the 30-meter pixel containing the home address and average NDVI in a buffer of 500 meters.

#### Precipitation

Total daily precipitation at the residential location was calculated using inverse distance weighting of ground monitors.

#### Daylight

Hours of daylight were calculated using the *sunriset* function in the *maptools* R package [33], which uses algorithms provided by the National Oceanic & Atmospheric Administration (NOAA) to calculate sunrise and sunset based on date and coordinates.

#### Geolocation

Space-time varying exposures were estimated using the location of the participant’s home at the time of the physical activity collection. Study personnel used a handheld GPS device to capture the latitude and longitude of the home of each participant who wore an accelerometer. The GPS results were manually reviewed and verified.

### Study population

We used data from PROGRESS (Programming Research in Obesity, GRowth, Environment, and Social Stress), a cohort based in Mexico City. PROGRESS is a longitudinal cohort study following mothers and their children since pregnancy. For our study, we focused on the children when they were 4–6 years old whose sedentary behavior was monitored within the years 2013 to 2015. Of the 609 total children in the cohort, we excluded 50 children who had missing covariate data and whose data did not meet the sedentary time guidelines, which is further described in the following section. After we applied this exclusion criteria, a total of 559 children remained in our study sample.

PROGRESS recruitment and follow-up procedures are briefly described next. Pregnant women were recruited in 2007–11 through the Mexican Social Security System (Instituto Mexicano del Seguro Social) and written informed consent was obtained. The eligibility criteria for enrollment into the study included the following: ≥ 18 years of age, ≤ 20 weeks gestation, no medical history of heart and kidney disease, no daily alcohol consumption, and no steroid or anti-epilepsy medication use. 1,054 pregnant women were initially enrolled and a total of 948 were followed up until delivery of a liveborn infant. Both mothers and their children have been followed since then at the Mexican National Institute of Perinatology (Instituto Nacional de Perinatología). Institutional Review Boards at the Icahn School of Medicine at Mount Sinai and at the Mexican National Institute of Public Health (Instituto Nacional de Salud Pública) approved the study procedures.

#### Sedentary time: Data collection and cleaning procedures

PROGRESS children wore an Actigraph GT3x+ accelerometer (Actigraph, Pensacola, FL) on their non-dominant wrist for approximately one week during the years 2013 to 2015. An accelerometer was secured to the child’s wrist by a field team member using a secure closure plastic wristband that could only be removed by cutting the wristband and participants were instructed to wear it at all times. There is a trade-off to placing accelerometers on wrists rather than other parts of the body, such as the waist. Wrist-worn monitors generally represent arm movements rather than whole body behavior [34]. However, placement on the waist may overestimate children’s sedentary time [35] and participant compliance among children is more likely for wearing an accelerometer on the wrist than on the hip [34], particularly for our study in which we expected participants to wear the monitors continuously for multiple days. Actigraph accelerometers are widely used for measuring objective levels of physical activity [12, 36, 37].

The Actigraph GT3x+ stored timestamps and counts along the x, y, and z axes for every 10-second epoch. We downloaded and processed the raw data to calculate the proportion of awake time each child spent being sedentary for each day. The formula is as follows:

$$Y_{ij}=\frac{S_{ij}}{T_{ij}}$$

where *Y*_*ij*_ is percent sedentary time on day *i* for participant *j*;

*S* is total sedentary time when awake;

*T* is total awake time

**Fig 1:**
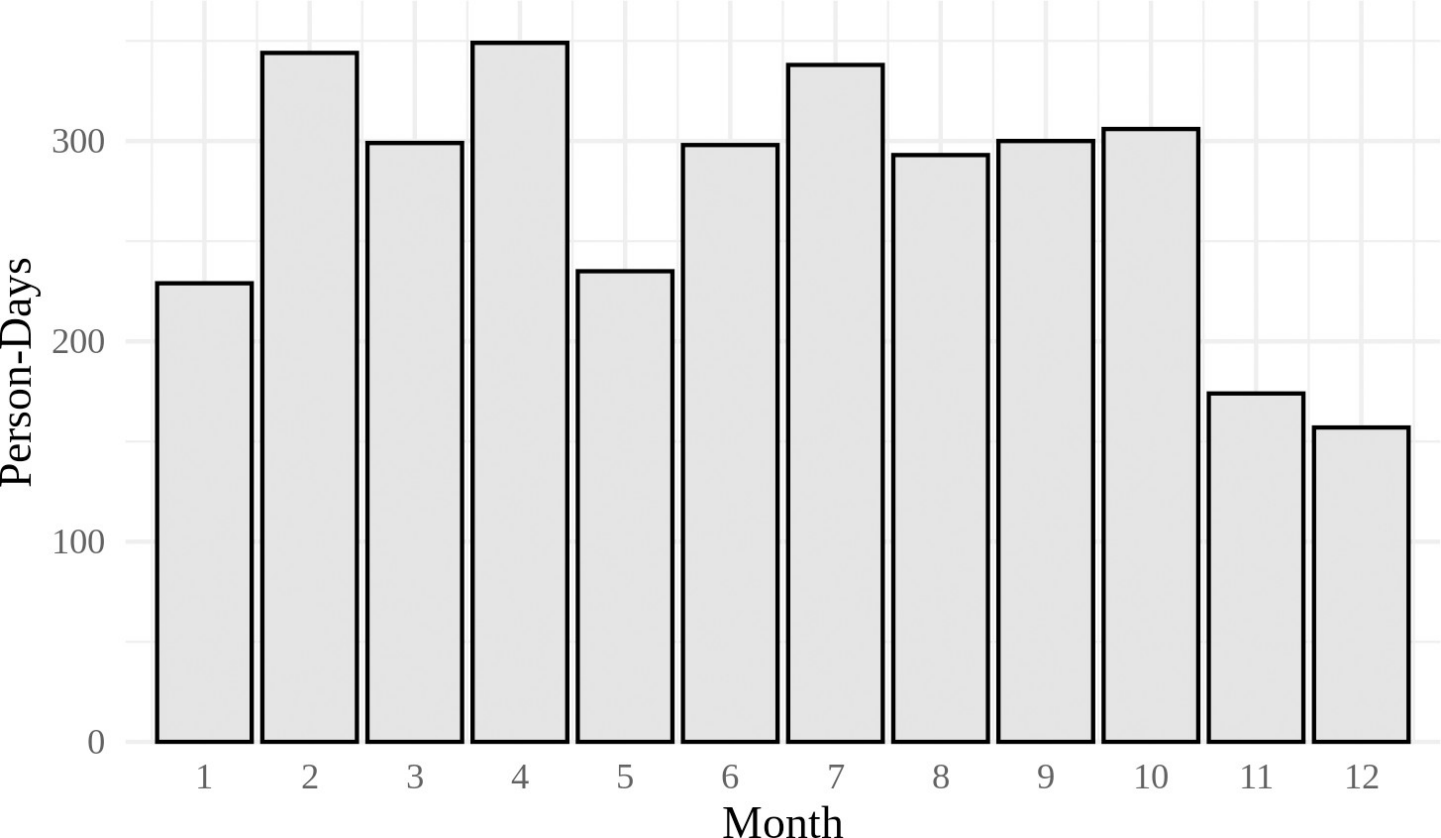
Histogram of accelerometer data collected by month of the year, in person-days (n = 3322).

Accelerometer data were collected throughout the year from 2013 to 2015. Fig 1 illustrates the distribution of data (in person-days) collected by month, beginning from January and ending in December. While data were collected during each month, the distribution is a bit uneven, with less data collected from November to January relative to the rest of the year. December had the fewest collection days (157 days), while April had the highest (349 days).

To process the data, we first converted the raw data to R file formats using the *acc* package [38]. Then, we used the *PhysicalActivity* package [39] to flag spurious time, which is non-wear time or the time when the accelerometer was not recording counts. We defined spurious time as 20 consecutive minutes of zero x-axis counts, a common threshold for children [40]. We also considered three other definitions of spurious time appropriate for our age group because different wear time algorithms may change the results [41]: 30 consecutive minutes of zero x-axis counts, 20 consecutive minutes of zero vector magnitude counts, and 30 consecutive minutes of zero vector magnitude counts. Vector magnitude is a common triaxial measurement of physical activity and it is calculated with the following equation: $\sqrt{x^{2}+y^{2}+z^{2}}$.

We then created custom functions in R version 3.4.4 for the following processing steps. We removed spurious time and sleep time [42–44] since inclusion of these times would overestimate real sedentary time. What remained was awake time, from which we differentiated sedentary time from time spent doing physical activity. We used validated thresholds from Johansson and colleagues [45], specifically a cut-point of ≤ 356 x-axis counts per 10-second epoch. Then, we calculated total sedentary time while awake for each day. This is a standard protocol validated by observational methods [45, 46]. However, 5-second intervals are recommended for young children to better characterize and capture short and different levels of activity [45, 46], but our finest temporal resolution is 10 seconds, so that is what we used. Following other standard procedures in the literature, we kept participant data if they met a threshold of ≥ 600 minutes of recorded activity for each day, had ≥ three weekdays and ≥ one weekend day [40]. Finally, we divided total sedentary time over total time while awake per day to produce our outcome of interest: percent daily sedentary time.

We used a wrist-specific cut-point that was calibrated by Johansson and colleagues for our children’s age group [45]. The threshold for sedentary time was calibrated using wrist measurements against the Children’s Activity Rating Scale (CARS) observational method. An age-specific threshold is needed for children in different age groups because children’s motor skills develop quickly when they are young and arm movements slow down as children get older [45]. Many previous studies did not use validated age-appropriate cut-points [35]. Our study does and in doing so incorporates a more reliable cut-point.

**Fig 2:**
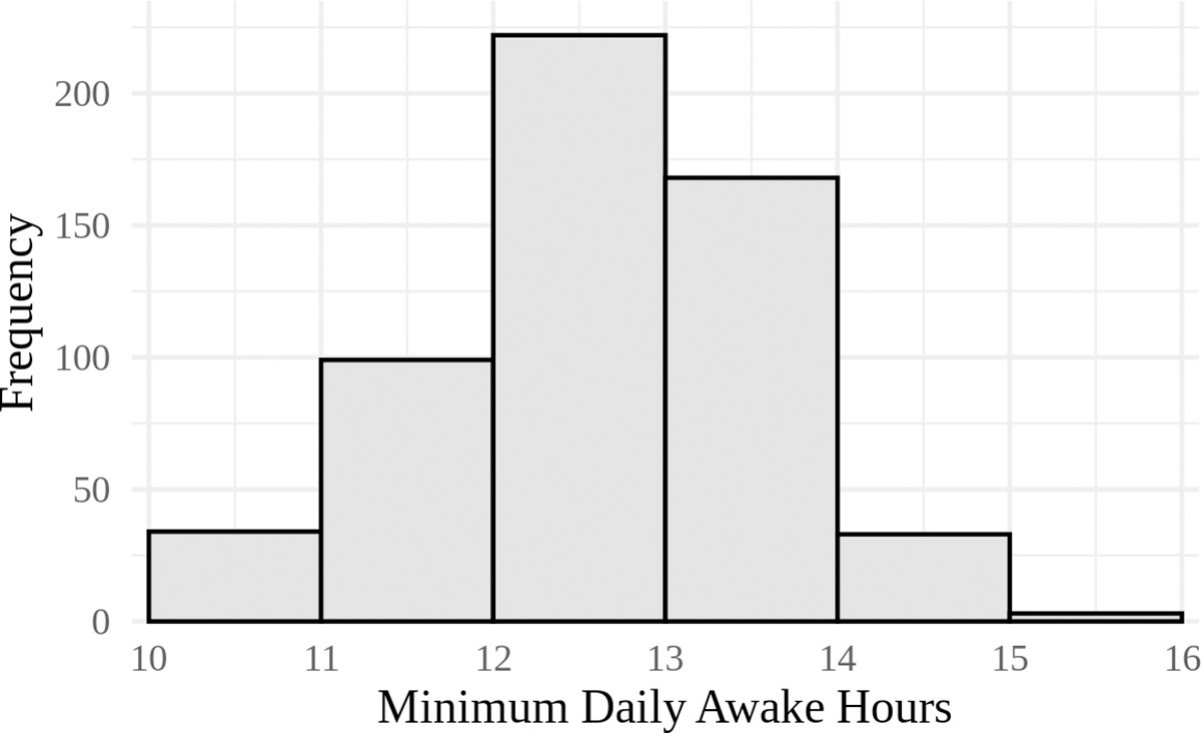
Histogram of minimum daily awake hours per child (n = 559).

To help readers assess whether the threshold of ≥ 600 minutes (10 hours) for total daily awake hours is appropriate, Fig 2 is provided below to illustrate the distribution of the minimum daily awake hours per child. Minimum daily awake hours are the lowest number of hours in a day that a child was awake during the monitoring period. These are days that met the 10-hour threshold for inclusion in our data analysis. In our sample, we have a total of 160 days of recorded awake time between 10–12 hours, which is approximately 5% of all days included in our analysis. Of these 160 days, there are 133 unique children, which represents 24% of all children included in our study. Other researchers have used a higher threshold of ≥ 720 minutes (12 hours) and five days for data inclusion [47], and we consider this alternative threshold as well.

#### Individual covariates

*Demographics*. Data on child’s age, sex, and maternal education were collected using a standardized questionnaire.

*BMI*. Weight and standing height were measured to calculate BMI for each child. Children’s BMI z-scores were calculated using the World Health Organization’s (WHO) guidelines [48].

*Sleep*. Sleep duration was calculated in ActiLife v6.11.9 using the Sadeh sleep algorithm for accelerometer data [49]. Sleep diaries completed by children’s parents were used to confirm whether intervals of low physical activity in the afternoon were nap times. More details on the collection and processing of covariate data are published elsewhere [42–44].

*School Days*. School days and holidays were identified using school calendars prescribed by the Mexican Secretariat of Public Education (Secretaría de Educación Pública). School holidays were combined with weekends to differentiate from school weekdays.

### Statistical analysis

We used generalized additive models (GAMs) to allow for nonlinear relationships between the dependent and independent variables [50]. We used GAMs to analyze the association of daily percent sedentary time as the outcome with individual characteristics (child age, sex, BMI z-score, sleep; maternal education; and school day versus holiday or weekend) and environmental exposures (temperature, NDVI, daylight, rain) as predictors. The temporal scale of our analysis is a day. The formula is as follows:

$$Sedtime_{ij}=(\alpha +u_{j})+\beta x_{ij}+f(age_{j})+f(rain_{ij})+f(bmi_{j})+f(day_{ij})+f(location_{j})$$

where *Sedtime*_*ij*_ is percent sedentary time on day *i* for participant *j*;

α and *u*_*j*_ are fixed and random intercepts;

*β* is *k*×1 vector of linear regression coefficients representing *n*×*k* matrix of covariates, *x*_*ij*_;

*f*(*age*_*j*_) is a thin plate regression spline for age of participant *j*;

*f*(*rain*_*ij*_) is a thin plate regression spline for rain on day *i* for participant *j*;

*f*(*bmi*_*j*_) is a thin plate regression spline for the BMI z-score of participant *j* in the GAM adjusting for diurnal variation, otherwise it is linear;

*f*(*day*_*ij*_) is a cyclic cubic regression spline for day of the year;

*f*(*location*_*j*_) is a thin plate regression spline of longitude and latitude

GAMs were modeled using the *mgcv* R package [51]. To implement *f*(*day*_*ij*_) in *mgcv*, we used *s*(*day*,*k* = 10,*bs* = "cc"), where day of the year was a numeric variable, the smooth term was specified using the *s* term and cubic spline basis, and the *k* = 10 default basis dimensions were used since changes to *k* did not change the results. To carry out *f*(*location*_*j*_) in *mgcv*, we used *s*(*x*,*y*), where *x* is longitude and *y* is latitude.

Four separate GAMs were modeled for maximum temperature, mean temperature, minimum temperature, and diurnal variation (maximum minus minimum). For each GAM, the temperature metric differed but all other variables remained the same. The covariates included child’s sex, age, BMI z-score, minutes of sleep; exposures to daily temperature, NDVI, hours of daylight, mean precipitation; maternal education; and weekend or holiday versus school day. All environmental exposures (i.e., temperature, NDVI, daylight, and precipitation) were concurrent with children’s daily activity levels. We also considered socioeconomic status, but since it was correlated with education and was not a significant covariate, we dropped socioeconomic status from the final models. All GAMs included participant-level random intercepts to account for repeated measures of sedentary time by day. We considered participant-level random slopes, but the GAM results were very similar and we decided to keep the simpler models with random intercepts alone. Since children were more active during the summer than in the winter, the models included a cyclic penalized cubic regression spline for day of the year to account for non-linear seasonal patterns in sedentary time and to isolate the effects of temperature. To account for spatial dependence, the models included a thin plate regression spline for the spatial location (longitude and latitude) of each participant’s residence. All predictors were checked for multicollinearity and non-linearity. Predictors were included as linear terms when their estimated degrees of freedom approximated one and the Akaike information criterion (AIC) of the GAMs did not improve with non-linear terms.

#### Hypothesis tests

To test our hypothesis that there is a curvilinear relationship between temperature and sedentary time, we first specified a smooth or linear term for maximum temperature, mean temperature, minimum temperature, and diurnal variation in separate GAMs. We then used AIC to compare GAMs with a smooth term versus a linear term for each temperature metric.

#### Sensitivity analyses

We conducted sensitivity analyses to determine how differences in variable coding impacted our results. First, we compared our temperature model predictions with temperature data from the nearest ground station and mean temperature across all of Mexico City using SIMAT (Mexico City’s Atmospheric Monitoring System) stations, which is data that is easier to obtain by a layperson.

Second, to examine whether results were sensitive to the choice of buffer size, we compared mean NDVI in a 250-meter buffer with a 30-meter NDVI pixel containing the home location and mean NDVI in a 500-meter buffer. Third, to analyze whether different physical activity processing decisions influenced our results, we compared percent sedentary time when classified using x-axis counts and 20 minutes of spurious time to x-axis counts and 30 minutes of spurious time; vector magnitude and 20 minutes of spurious time; and vector magnitude and 30 minutes of spurious time.

Finally, we assessed whether our results changed with stricter data inclusion criteria (i.e., a minimum of 12 hours per day and 5 total days). We calculated differences in estimated coefficients and standard errors, and examined whether the null hypothesis was similarly rejected or not rejected.

## Results

**Fig 3:**
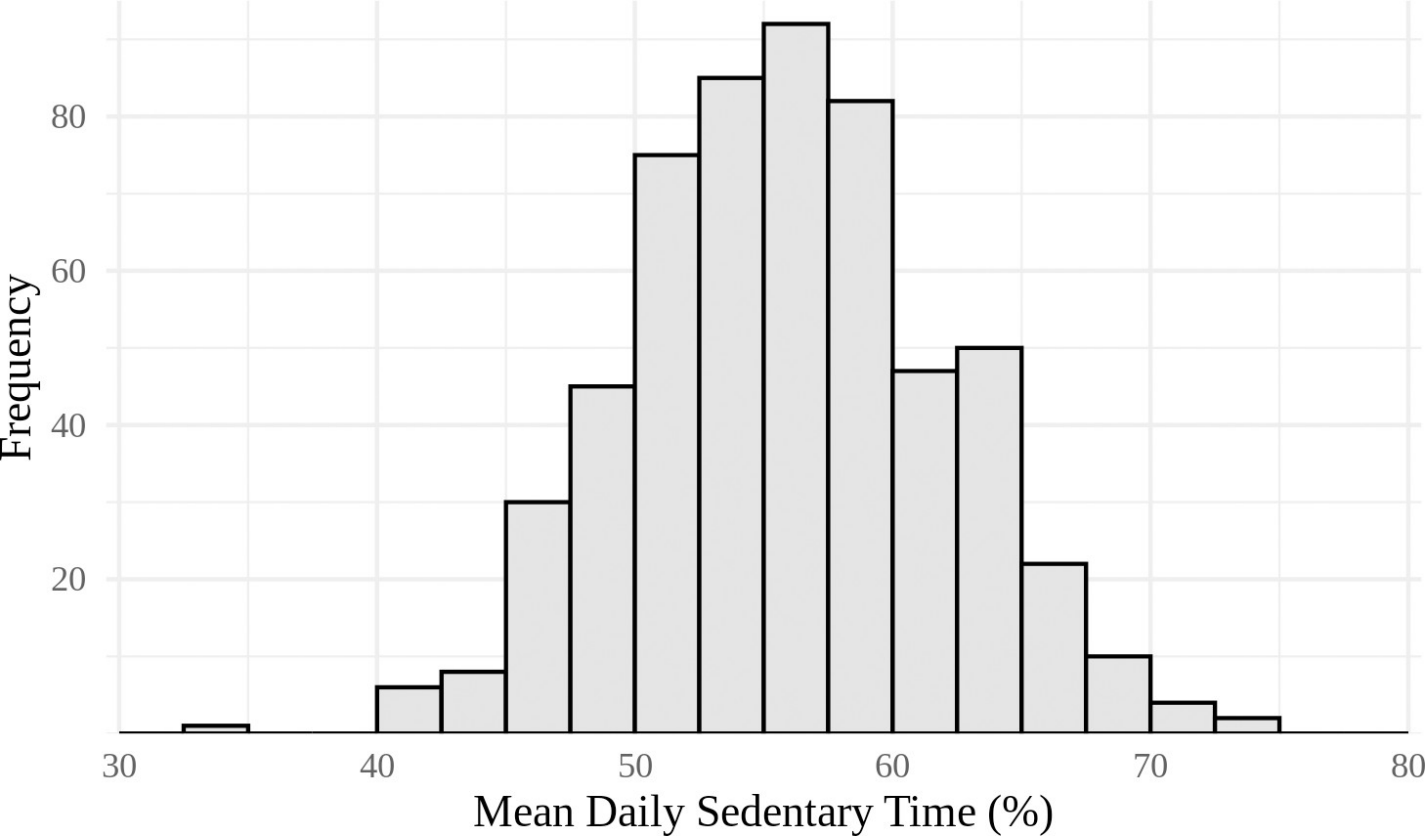
Histogram of mean daily sedentary time per child as a percent of total time awake (n = 559).

Table 1 summarizes the demographic characteristics, environmental exposures, and sedentary time of the cohort. The ratio of male to female children is nearly equal, with males comprising 51% of the sample. Most mothers (60%) have a high school education or more and children’s mean age is 4.8 years. The average temperature is 22.1°C for daily maximum temperature, 15.4°C for daily mean temperature, 10.2°C for daily minimum temperature, and 11.9°C for diurnal variation. On average, most children spend more than 50% of their tracked waking hours being sedentary (an average of seven hours and 54 minutes). While this is typical for children in this age group, it still represents an overly large proportion of sedentary time during waking hours [52, 53]. Fig 3 illustrates the mean daily sedentary time for each child.

**Table 1:** Summary statistics.

Variable	Mean	SD
*Outcome*		
Daily Sedentary Time (%)	56.0	6.0
*Environmental Exposures*		
Daily Maximum Temperature (°C)	22.1	2.5
Daily Mean Temperature (°C)	15.4	2.2
Daily Minimum Temperature (°C)	10.2	2.4
Daily Diurnal Variation (°C)	11.9	2.3
Mean NDVI (250-m buffer)	0.19	0.08
Daily Daylight (hours)	12.2	0.8
Daily Total Precipitation (mm)	2.8	3.6
*Cohort Characteristics*		
Age (years)	4.8	0.5
BMI Z-Score	0.2	1.1
Daily Sleep (minutes)	470	72
** **	**N**	**%**
*Sex*		
Male	285	51
Female	274	49
*Maternal Education*		
Less than High School	223	39.9
High School	204	36.5
More than High School	132	23.6
*Type of Day*		
School Day	1675	50.4
Holiday or Weekend	1647	49.6

We reject our hypothesis that there is a curvilinear relationship between temperature and sedentary time. In all GAMs with non-linear terms for maximum temperature, mean temperature, minimum temperature, and diurnal variation, the estimated degrees of freedom is 1. The AICs comparing GAMs with non-linear versus linear predictors are nearly the same. The AICs for a GAM with a smooth versus linear temperature covariate are 22238.33 and 22238.32 for maximum temperature, 22245.96 and 22245.98 for mean temperature, 22257.63 and 22257.61 for minimum temperature, and 22242.94 and 22242.88 for diurnal variation, respectively.

Tables 2 and 3 present the results of four GAMs, with each model including a different daily temperature measure as a predictor. Model 1 includes maximum temperature, Model 2 includes mean temperature, Model 3 includes minimum temperature, and Model 4 includes diurnal variation. We have a separate model for each type of temperature rather than one model that includes all four because they are highly correlated with one another.

**Table 2:** GAM results with daily percent sedentary time as the outcome.

	*Model 1*: *Maximum Temperature*			*Model 2*: *Mean Temperature*			*Model 3*: *Minimum Temperature*		
Variable	Estimate	95% CI	P-Value	Estimate	95% CI	P-Value	Estimate	95% CI	P-Value
(Intercept)	77.9	69.6, 86.2	< 0.001	75.4	67.1, 83.7	< 0.001	77.4	68.8, 86.0	< 0.001
Daily Temperature (°C)	-0.26	-0.39, -0.13	< 0.001	-0.27	-0.45, -0.09	0.004	0.06	-0.12, 0.23	0.54
Mean NDVI (250-m buffer)	0.0004	-6.94, 6.94	1.00	0.26	-6.74, 7.27	0.94	3.09	-3.78, 9.97	0.38
Daily Daylight (hours)	-1.20	-1.90, -0.50	< 0.001	-1.12	-1.87, -0.38	0.003	-1.73	-2.49, -0.97	< 0.001
Daily Sleep (minutes)	-0.004	-0.008, -0.001	0.02	-0.004	-0.008, -0.001	0.01	-0.004	-0.008, -0.001	0.02
BMI Z-Score	-0.17	-0.62, 0.29	0.47	-0.16	-0.62, 0.29	0.48	-0.17	-0.62, 0.29	0.47
Sex (Ref: Male)	0.21	-0.77, 1.19	0.67	0.20	-0.78, 1.18	0.69	0.14	-0.84, 1.11	0.79
Maternal HS Education (Ref: LHS)	0.42	-0.71, 1.55	0.46	0.43	-0.70, 1.56	0.46	0.40	-0.72, 1.53	0.48
Maternal More than HS Education (Ref: LHS)	2.41	1.13, 3.69	< 0.001	2.39	1.11, 3.67	< 0.001	2.33	1.06, 3.61	< 0.001
Holiday/Weekend (Ref: School Day)	-0.42	-0.91, 0.07	0.09	-0.45	-0.95, 0.04	0.07	-0.37	-0.87, 0.13	0.14
s(Daily Total Rain) (mm)	0.03	0.01, 0.11	0.001	0.04	0.01, 0.12	< 0.001	0.04	0.01, 0.13	< 0.001
s(Age) (years)	3.29	0.74, 14.6	0.09	3.43	0.82, 14.5	0.08	3.56	0.86, 14.7	0.07
s(Day of Year)	7.3 x10^-5^	3.9 x10^-14^, 1.4 x10^5^	0.60	8.8 x10^-5^	3.6 x10^-12^, 2.2 x10^3^	0.71	6.6 x10^-5^	2.3 x10^-14^, 1.9 x10^5^	0.90
s(Participant)	5.21	4.84, 5.62	< 0.001	5.21	4.83, 5.61	< 0.001	5.18	4.81, 5.59	< 0.001
s(x,y)	0.67	6.7 x10^-13^, 6.8 x10^11^	0.20	0.91	2.6 x10^-9^, 6.8 x10^8^	0.23	0.64	7.7 x10^-17^, 5.4 x10^15^	0.52
Adjusted R^2^	0.42			0.42			0.41		

Abbreviation: LHS, Less than High School.

**Table 3:** GAM results continued with daily percent sedentary time as the outcome.

	*Model 4*: *Diurnal Variation (Max—Min)*		
Variable	Estimate	95% CI	P-Value
(Intercept)	80.6	72.0, 89.1	< 0.001
Daily Temperature (°C)	-0.23	-0.35, -0.12	< 0.001
Mean NDVI (250-m buffer)	2.01	-4.78, 8.80	0.56
Daily Daylight (hours)	-1.71	-2.38, -1.03	< 0.001
Daily Sleep (minutes)	-0.004	-0.008, -0.001	0.02
Sex (Ref: Male)	0.19	-0.79, 1.16	0.71
Maternal HS Education (Ref: LHS)	0.41	-0.72, 1.53	0.48
Maternal More than HS Education (Ref: LHS)	2.41	1.14, 3.69	< 0.001
Holiday/Weekend (Ref: School Day)	-0.33	-0.83, 0.16	0.19
s(Daily Total Rain) (mm)	0.03	0.01, 0.10	0.006
s(Age) (years)	3.35	0.69, 16.2	0.10
s(BMI Z-Score)	0.49	0.04, 5.84	0.23
s(Day of Year)	8.9 x10^-5^	9.8 x10^-14^, 8.2 x10^4^	0.36
s(Participant)	5.19	4.81, 5.59	< 0.001
s(x,y)	1.27	1.1 x10^-8^, 1.4 x10^8^	0.38
Adjusted R^2^	0.42		

Abbreviation: LHS, Less than High School.

Across the four GAMs, there are differences in the between and within participant variability in sedentary time by temperature type. The F-values are 15.2 for maximum temperature, 8.3 for mean temperature, 0.38 for minimum temperature, and 15.5 for diurnal variation. (For GAMs with temperature as the outcome variable, the F-values for sedentary time as the covariate are 17.8, 10.8, 0.59, and 16.6 for maximum, mean, minimum, and diurnal variation, respectively.)

In Tables 2 and 3, the estimates for the random parameters are the variance components for each parameter. Participant-level random intercepts are significant at the threshold of p<0.05 and their coefficient estimates are higher relative to other model parameters in all GAMs. An estimated 5.2% (95% CI: 4.8 to 5.6%) of the between and within variation in sedentary time is explained by participant-level random intercepts. Total precipitation has a significant (p<0.001) association with sedentary time. This association is positive and approximately linear from 0 to 20 mm, and it accounts for the vast majority of observations. There is not enough compelling support for the downward trend after 20 mm. Only 1.9% of the data is above 20 mm and there is greater uncertainty. The non-linear terms for age, day of the year, and residential location are not statistically significant. In the GAM with diurnal variation as a predictor, BMI z-score is a non-linear term but it is not statistically significant.

Within the range of temperatures observed during the study, daily maximum temperature has a significant, negative linear relationship with daily sedentary time (Table 2, Model 1, p<0.001). For each 1°C higher maximum temperature, children have on average 0.26% less sedentary time (95% CI: -0.39 to -0.13%). Each 1°C higher maximum temperature approximates 2.2 fewer minutes of sedentary time on average (ranging from 1.6 to 3.0 minutes) for the children in our study.

Other significant covariates that have a negative linear relationship with daily sedentary time are hours of daylight (p<0.001, 95% CI: -1.90 to -0.50%) and minutes of sleep (p = 0.02, 95% CI: -0.008 to -0.001%). Compared to those with mothers with less than high school education, children of mothers with more than high school education have higher levels of sedentary time on average (p<0.001, 95% CI: 1.13 to 3.69%).

**Fig 4:**
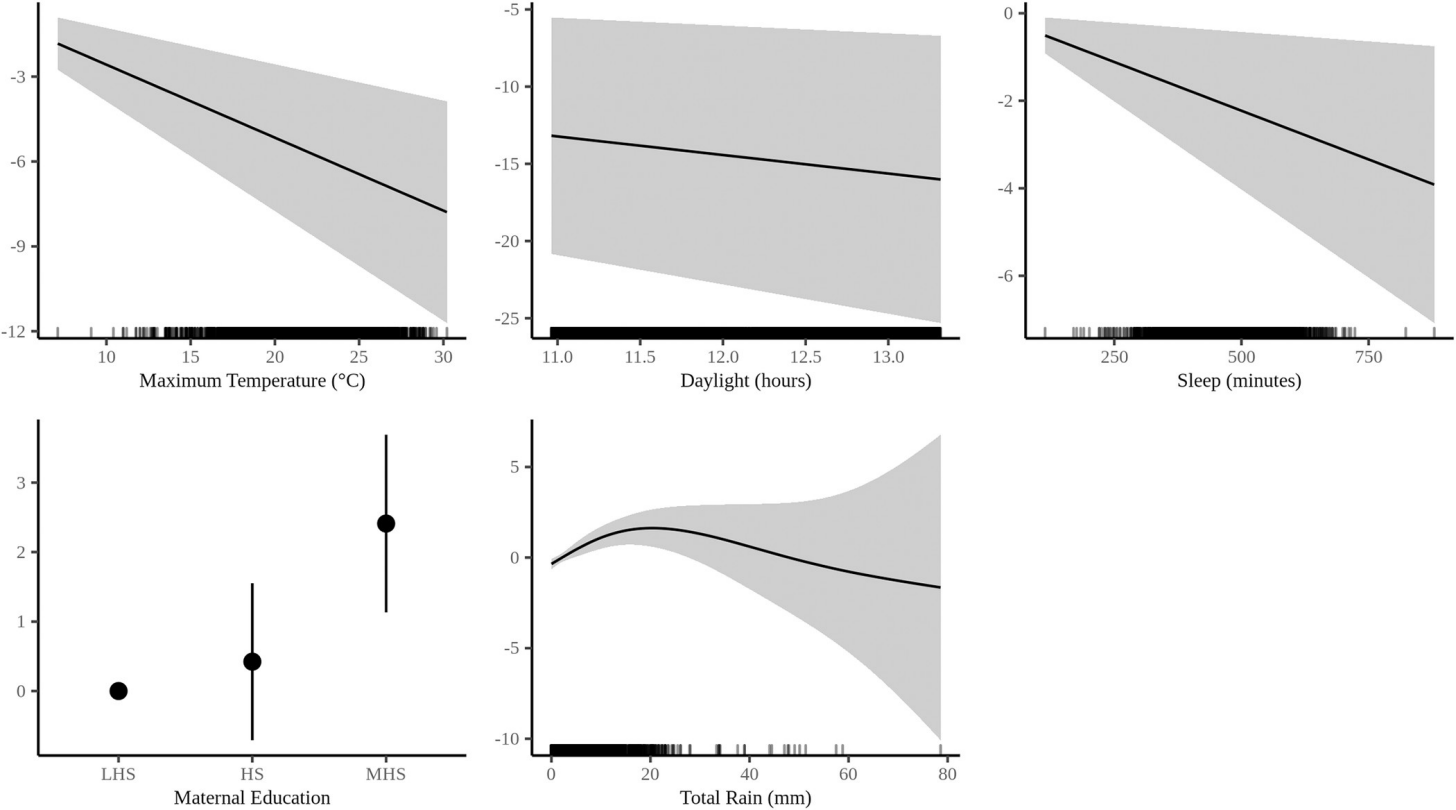
Maximum temperature GAM covariate plots with predicted daily percent sedentary time along y-axis.

Fig 4 depicts the plots for each statistically significant covariate for the GAM with daily maximum temperature. Each plot visualizes the effect estimate for each statistically significant term from the multivariable model, whereby the y-axis is predicted daily percent sedentary time and the gray area represents the 95% CI for the respective term. A higher y-axis value means more sedentary time and less physical activity. S1–S3 Figs (Model 1) include all covariate plots.

The results for the GAM with daily mean temperature (Table 2, Model 2) are similar to the results for daily maximum temperature. Daily mean temperature (95% CI: -0.45 to -0.09%), hours of daylight (95% CI: -1.87 to -0.38%), and minutes of sleep (95% CI: -0.008 to -0.001%) have significant negative linear associations with proportion of time spent being sedentary. Children have on average 0.27% less sedentary time for each 1°C higher mean temperature. Each 1°C higher mean temperature approximates 2.3 fewer minutes of sedentary time on average, with a range of 1.6 to 3.1 minutes. Children of mothers with more than high school education have higher levels of sedentary time. S1–S3 Figs (Model 2) depict the plots for each covariate.

The statistically significant covariates in the GAM results with diurnal variation (Table 3) are also analogous to the ones in the GAMs with mean and maximum temperatures. The following variables have significant negative linear correlations with sedentary time: diurnal variation (95% CI: -0.35 to -0.12%), daylight (95% CI: -2.38 to -1.03%), and sleep (95% CI: -0.008 to -0.001%). Children spend on average 0.23% less sedentary time for each 1°C higher diurnal variation. Each 1°C higher mean temperature approximates 2.0 fewer minutes of sedentary time on average, with a range of 1.4 to 2.7 minutes. Children of mothers with more than high school education have significantly higher levels of sedentary time (95% CI: 1.14 to 3.69%). S1–S3 Figs (Model 4) depict the plots for each covariate.

The GAM results with daily minimum temperature (Table 2, Model 3) are distinct from the results for daily maximum and mean temperatures. Daily minimum temperature does not have a significant association with daily sedentary time. The linear covariates that do have statistically significant associations similar to the other GAM results are hours of daylight (95% CI: -2.49 to -0.97%), minutes of sleep (95% CI: -0.008 to -0.001%), and mothers with more than high school education (95% CI: 1.06 to 3.61%). S1–S3 Figs (Model 3) illustrate the covariate plots.

We generally find consistent results when comparing the multivariable GAMs to univariable versions. To assess the individual association of each continuous variable with percent daily sedentary time, GAMs were generated with percent daily sedentary time as the outcome variable, a participant-level random intercept to account for repeated measures of sedentary time, and a continuous variable as the independent variable. The continuous variables included temperature (maximum, mean, minimum, and diurnal variation), NDVI, daylight, rain, sleep, age, and BMI z-score. Each was assessed for non-linearity. S1 and S2 Tables summarize these results. Predictors were considered linear when their estimated degrees of freedom approximated one and the AIC of the GAM did not improve with a non-linear term. Statistically significant linear associations with sedentary time were identified for maximum temperature, mean temperature, daylight, and sleep. Statistically significant non-linear associations with sedentary time were found for diurnal variation and precipitation, however, most observations for both followed an approximately linear association. Diurnal variation followed a mostly negative association while precipitation was a mostly positive association with sedentary time. BMI z-score was linear in the univariable GAM while non-linear in the multivariable GAM adjusting for diurnal variation; however, BMI z-score was consistently not statistically significant.

We also find consistent results in our sensitivity analyses. We compared temperature model predictions to temperature data from the nearest ground station and mean temperature from SIMAT; the 30-meter NDVI pixel containing the home location, mean NDVI in a 250-meter buffer, and mean NDVI in a 500-meter buffer; and percent sedentary time when classified using x-axis counts or vector magnitude and 20 or 30 minutes of spurious time. All results are similar in significance and magnitude. When comparing the results using stringent data inclusion criteria (12-hour days, 5 days a week) to the original results (10-hour days, 4 days a week), we find negligible differences. The differences in the estimated coefficients (12-hour/5 days minus 10-hour/4 days) are 0.009 for maximum temperature, 0.0002 for mean temperature, -0.044 for minimum temperature, and 0.025 for diurnal variation (all coefficient signs were the same). The differences in standard errors are 0.007 for maximum temperature, 0.009 for mean and minimum temperature, and 0.006 for diurnal variation. The null hypothesis is rejected for maximum, mean, and diurnal variation, but not rejected for minimum temperature, and this is the case for both models with different data inclusion criteria. S3 Table contains the results when using mean temperature from SIMAT.

## Discussion

Our study’s main findings are that daily maximum temperature, mean temperature, and diurnal variation are negatively and linearly associated with all-day sedentary time while daily minimum temperature does not have a significant association for young children in Mexico City. In other words, higher maximum and mean temperatures, as well as greater diurnal variation, are linked to more physical activity. Our findings differ from our hypothesis, in which we expected the relationship between temperature (maximum, mean, minimum, and diurnal variation) and sedentary time to be curvilinear. Our results are likely due to the moderate temperature ranges for our observations. Generally, the temperature in Mexico City ranges from 3°C to 30°C and it is extremely rare for the temperature to be outside of these bounds.

Our results add to the growing body of research on the relationship between temperature and sedentary time by including research on a middle-income population with distinctive social norms and infrastructural resources related to physical activity located in a city with a temperate climate. We find that small variations within a relatively stable climate are correlated with differences in sedentary time. Our study in a temperate climate offers unique insights and additional evidence on how temperature is linked to sedentary behavior. It suggests that relative differences in temperature, as well as absolute differences, are important for understanding activity behaviors. In addition to examining both mean and maximum temperature’s relationship to sedentary time, we also use minimum temperature to explore the role of low temperature and diurnal variation to assess temperature variation. In our study, we do not find an association between minimum temperature and sedentary time. This may be because daily minimum temperatures often occur at night when children are more likely to be asleep and our temperature range does not include subfreezing temperatures.

For all GAMs, four covariates have a consistent significant association with daily percent sedentary time: daylight, sleep, more than high school education, and precipitation. Hours of daylight have a negative relationship with daily sedentary time, suggesting that longer daylight hours offer more time and opportunities for children to play outside in our multivariable model adjusted for seasonality, temperature, and precipitation. Minutes of sleep are negatively associated with daily sedentary time. The restorative qualities of sleep may be important for promoting physical activity among children. Total precipitation has a significant, positive, approximately linear association with daily sedentary time, demonstrating that rain is a strong deterrent to physical activity. Rain may compel kids to remain indoors and be inactive. Children of mothers with more than high school education have higher levels of sedentary time compared to those of mothers with less than high school education. The children of mothers with more education may have greater access to cars reducing active transport; as well as more access to digital devices that encourage sedentary behaviors.

The current scientific evidence on correlates of sedentary time among young children is inconsistent and thin, with particularly limited research on season and related factors [12]. In a recent systematic review on correlates of sedentary time among young children, Pereira and colleagues found inconsistent associations for four correlates (child sex, sleep, childcare versus non-childcare, and childcare type), no association with 39 correlates, and 41 correlates that had been infrequently analyzed [54]. Of the existing evidence relevant to our study, the research suggests that: adverse weather conditions such as fewer daylight hours and more rain are linked with less activity [8, 13]; BMI has a positive association with sedentary behaviors [7, 12] or no association [54]; the relationship between sleep habits and sedentary time is undetermined [54]; sedentary behavior increases with age [55]; and that an increase in residential NDVI is associated with less excessive sedentary behaviors [56]. Our study’s results add to this evidence base by finding that daylight and sleep have significant, negative, linear associations with sedentary time; rain has an approximately positive linear association; and children of mothers with more than high school education have higher levels of sedentary time on average compared to children of mothers with less than high school education.

Our results demonstrate that children in Mexico City are more likely to be active in warmer weather which suggests a need for interventions that discourage sedentary behavior when it is cooler outside. If we rescale the effect estimates from 1°C to 10°C in temperature, then children could potentially spend 22 to 23 fewer minutes being sedentary with each 10°C increase in maximum and mean temperature, respectively. For many children, 22 to 23 minutes being active instead of sedentary has the potential to make a considerable difference to their daily routine and to their overall health and fitness. Our sample includes children 4–6 years old, an age range that overlaps with two different physical activity guidelines in the U.S.–one for children 3–5 years old and the other for children and adolescents 6–17 years old [57]. The suggested guidelines are a minimum of 3 hours of any activity per day for children 3–5 years old and 60 minutes of MVPA per day for children 6–17 years old [57]. Most children in our sample are already active for at least 3 hours a day and our model predicts that a 10 degree increase in maximum temperature results in a small increase of 4 days (representing 4 unique children) meeting this guideline. If we extend the MVPA guideline to younger children and assume that sedentary time transforms to MVPA time, then many more children are predicted to meet the MVPA guideline given a 10 degree increase in maximum temperature, specifically 61 kids totaling 74 days. Of these, there are 28 unique kids over 5 years old, who together represent a total of 36 days. Appropriate interventions to encourage physical activity may include indoor play spaces that shield against adverse weather conditions. There may also be a need for active transport, as parents with vehicles are more likely to drive their children to their destinations than to navigate Mexico City’s unreliable and fragmented public transit system.

While the focus of our paper is on the relationship between temperature and sedentary time, we should note that our paper also extends the work of previous studies on green space and physical (in)activity by including neighborhood greenness in our models and finding a non-significant association between greenness and sedentary time in a middle-income urban population. It is more common for studies to take place in developed countries like Europe, Canada, and Australia [10, 58–60]. Additionally, the results from earlier studies are varied, with some observing a positive association between green space and physical activity [10, 61], negative association with excessive screen time [58], and no association [56, 62]. To our knowledge, this is also the first research endeavor in Mexico to consider objectively measured green space in conjunction with sedentary time rather than subjective measures that are susceptible to response bias and memory recall issues. More research is needed to better understand the role of green space in influencing how active and inactive people are in non-Western contexts. For instance, in Mexico City, many parks in working-class neighborhoods are considered unsafe, thus restricting outdoor opportunities for physical activity.

Our study’s strengths include the use of objective measures of sedentary time and meteorological exposures at high spatiotemporal resolutions. There are inconsistent results in the limited research on sedentary behavior and temperature, partly due to differing methods for measuring sedentary behavior and weather [8, 63]. Physical activity and sedentary behavior are more likely to be assessed subjectively through surveys and thus subject to issues of memory recall and response bias, while season is more likely to be utilized as a proxy for specific attributes like temperature [8, 63]. Seasonality is an inadequate and imprecise proxy for temperature. Our study supports this, as we find measures of temperature and not seasonality to be significant predictors of sedentary time. Furthermore, seasons can vary substantially between diverse parts of the world, making it difficult to compare results in different study sites [17, 18, 20]. In addition to using objective measures for both sedentary time and temperature, we carried out sensitivity analyses for both measurements to test the robustness of our models’ results and found the associations to remain stable.

We use GAMs to investigate the relationship between temperature and sedentary behavior, and our statistical methods offer a couple of advantages over simple linear multivariate regression models. First, GAMs allow us to account for repeated measures of sedentary time using penalized regression terms to estimate random effects, critical for capturing day-to-day fluctuations in activity behaviors that are unlikely to be independent of each other. Measures of sedentary behavior and physical activity are often aggregated over the entire period of observation, thus overlooking important temporal patterns. Second, GAMs allow flexible non-linear terms and non-linear relationships. In our study, we use a cyclic smoothing term for day of the year to isolate temperature variation from seasonality and to more precisely estimate our association of interest with temperature.

Although our study has multiple strengths, it also has several limitations. One is that we only have children’s residential locations. When we assign time-varying environmental exposures, we assume the exposures all took place at or near their place of residence, which is a reasonable assumption for young children. However, it is possible that our participants may spend some time elsewhere. Since we do not know precisely where our participants spend their time, we cannot distinguish indoor from outdoor activities and we are uncertain that warmer weather actually encourages children to spend more time playing outside. Future research would benefit from GPS tracking of participants’ daily movements to better assign environmental exposures and identify spaces that discourage sedentary behaviors [64]. Another limitation is our use of NDVI as a measure of green space. Children’s outdoor activities may be shaped by parental concerns related to safety. In this case, access to safe playgrounds may be more important than how green their neighborhood is. An additional limitation is that our participants live in relatively close proximity to one another, resulting in limited spatial variation in environmental exposures. In the future, cohort studies should consider recruiting spatially heterogeneous participants to better investigate how spatially varying exposures influence health outcomes. For other researchers interested in conducting a similar study, we want to make transparent that studying young children can be more challenging than studying adults or the elderly. Children are a vulnerable study population and informed consent needs to be acquired from a child’s guardian. Furthermore, monitor wear compliance among young children is a concern [34, 45].

It remains important to measure sedentary time and to assess its correlates among young children as early childhood is a time period when they are developing rapidly and acquiring sedentary behaviors that may persist as they grow older [5, 6, 45, 65]. Our findings also provide a baseline for tracking sedentary behavior over time, which is an area of study in vital need of more research [65]. Going forward, future research endeavors should include longitudinal analyses at different life stages as an extension of our cross-sectional findings. With the PROGRESS cohort, we are collecting additional accelerometer data as the children get older and we can examine how physical activity levels change over time with respect to temperature and other environmental exposures. It is critical to capture activity levels during the early childhood years as this is a life-stage before there is much autonomy in play behavior. We can also investigate whether sedentary children are more likely to remain sedentary as they get older. Qualitative methods should be used in conjunction with quantitative methods to better understand the underlying processes driving the patterns identified.

Another future research prospect involves additional case studies. Other megacities with climates like that of Mexico City should be studied to assess the extent to which our findings can be generalizable to comparable megacities. More case studies should also take place in areas with different social customs related to physical activity that are located in geographically and climatologically diverse areas that experience more temperature variability than Mexico City, where the climate is moderate all year round. In other places with extremely hot and cold temperatures, we expect non-linear relationships between temperature and physical activity, and low activity at temperature extremes.

As we experience population increases in sedentary behavior and related chronic diseases, it becomes increasingly important to understand how environmental factors like temperature are linked to health behaviors and outcomes over the life course. Our project is particularly important for including and tracking the environmental exposures and physical activity levels of young children. In this age group, health behaviors are more flexible and amenable to change, offering critical opportunities for early intervention, mitigation of adverse environments, and healthy development.

## Supporting information

S1 Fig GAM covariate plots with predicted daily percent sedentary time along y-axis. (TIF)

S2 Fig GAM covariate plots with predicted daily percent sedentary time along y-axis. (TIF)

S3 Fig GAM covariate plots with predicted daily percent sedentary time along y-axis. (TIF)

S1 Table Univariable GAMs results to assess non-linearity. (DOCX)

S2 Table Univariable GAM results with daily percent sedentary time along y-axis. (DOCX)

S3 Table Additional GAM results adjusting for SIMAT mean temperature with daily percent sedentary time along y-axis. (DOCX)

## References

[1] de Rezende LFM, Rodrigues Lopes M, Rey-López JP, Matsudo VKR, Luiz O do C. Sedentary behavior and health outcomes: an overview of systematic reviews. PLoS One. 2014;9: e105620 10.1371/journal.pone.0105620 25144686 PMC4140795

[2] LeBlanc AG, Spence JC, Carson V, Connor Gorber S, Dillman C, Janssen I, et al Systematic review of sedentary behaviour and health indicators in the early years (aged 0–4 years). Appl Physiol Nutr Metab. 2012;37: 753–772. 10.1139/h2012-063 22765839

[3] Mitchell JA, Pate RR, Beets MW, Nader PR. Time spent in sedentary behavior and changes in childhood BMI: a longitudinal study from ages 9 to 15 years. Int J Obes. 2013;37: 54–60. 10.1038/ijo.2012.41 22430304

[4] Tremblay MS, LeBlanc AG, Kho ME, Saunders TJ, Larouche R, Colley RC, et al Systematic review of sedentary behaviour and health indicators in school-aged children and youth. Int J Behav Nutr Phys Act. 2011;8: 98 10.1186/1479-5868-8-98 21936895 PMC3186735

[5] Telama R, Yang X, Viikari J, Välimäki I, Wanne O, Raitakari O. Physical activity from childhood to adulthood: a 21-year tracking study. Am J Prev Med. 2005;28: 267–273. 10.1016/j.amepre.2004.12.003 15766614

[6] Acosta W, Meek TH, Schutz H, Dlugosz EM, Vu KT, Garland T Jr. Effects of early-onset voluntary exercise on adult physical activity and associated phenotypes in mice. Physiol Behav. 2015;149: 279–286. 10.1016/j.physbeh.2015.06.020 26079567

[7] Van Der Horst K, Paw MJCA, Twisk JWR, Van Mechelen W. A brief review on correlates of physical activity and sedentariness in youth. Med Sci Sports Exerc. 2007;39: 1241–1250. 10.1249/mss.0b013e318059bf35 17762356

[8] Tucker P, Gilliland J. The effect of season and weather on physical activity: a systematic review. Public Health. 2007;121: 909–922. 10.1016/j.puhe.2007.04.009 17920646

[9] Bancroft C, Joshi S, Rundle A, Hutson M, Chong C, Weiss CC, et al Association of proximity and density of parks and objectively measured physical activity in the United States: A systematic review. Soc Sci Med. 2015;138: 22–30. 10.1016/j.socscimed.2015.05.034 26043433

[10] McMorris O, Villeneuve PJ, Su J, Jerrett M. Urban greenness and physical activity in a national survey of Canadians. Environ Res. 2015;137: 94–100. 10.1016/j.envres.2014.11.010 25527908

[11] Foraster M, Eze IC, Vienneau D, Brink M, Cajochen C, Caviezel S, et al Long-term transportation noise annoyance is associated with subsequent lower levels of physical activity. Environ Int. 2016;91: 341–349. 10.1016/j.envint.2016.03.011 27030897

[12] Rich C, Griffiths LJ, Dezateux C. Seasonal variation in accelerometer-determined sedentary behaviour and physical activity in children: a review. Int J Behav Nutr Phys Act. 2012;9: 49 10.1186/1479-5868-9-49 22546178 PMC3511197

[13] Atkin AJ, Sharp SJ, Harrison F, Brage S, Van Sluijs EMF. Seasonal Variation in Children’s Physical Activity and Sedentary Time. Med Sci Sports Exerc. 2016;48: 449–456. 10.1249/MSS.0000000000000786 26429733 PMC4762193

[14] Ridgers ND, Salmon J, Timperio A. Seasonal changes in physical activity during school recess and lunchtime among Australian children. J Sports Sci. 2018;36: 1508–1514. 10.1080/02640414.2017.1398892 29094653

[15] Larouche R, Gunnell K, Bélanger M. Seasonal variations and changes in school travel mode from childhood to late adolescence: A prospective study in New Brunswick, Canada. Journal of Transport & Health. 2019 pp. 371–378. 10.1016/j.jth.2018.08.012

[16] Nilsen AKO, Anderssen SA, Ylvisaaker E, Johannessen K, Aadland E. Physical activity among Norwegian preschoolers varies by sex, age, and season. Scand J Med Sci Sports. 2019;29: 862–873. 10.1111/sms.13405 30740779

[17] Remmers T, Thijs C, Timperio A, Salmon JO, Veitch J, Kremers SPJ, et al Daily Weather and Childrenʼs Physical Activity Patterns. Medicine & Science in Sports & Exercise. 2017 pp. 922–929. 10.1249/MSS.0000000000001181 28060036

[18] Lewis LK, Maher C, Belanger K, Tremblay M, Chaput J-P, Olds T. At the Mercy of the Gods: Associations Between Weather, Physical Activity, and Sedentary Time in Children. Pediatr Exerc Sci. 2016;28: 152–163. 10.1123/pes.2015-0076 26098393

[19] Mattocks C, Leary S, Ness A, Deere K, Saunders J, Kirkby J, et al Intraindividual variation of objectively measured physical activity in children. Med Sci Sports Exerc. 2007;39: 622–629. 10.1249/mss.0b013e318030631b 17414799

[20] Harrison F, on behalf the ICAD collaborators, Goodman A, van Sluijs EMF, Andersen LB, Cardon G, et al Weather and children’s physical activity; how and why do relationships vary between countries? International Journal of Behavioral Nutrition and Physical Activity. 2017 10.1186/s12966-017-0526-7 28558747 PMC5450267

[21] Matthews CE, Freedson PS, Hebert JR, Stanek EJ III, Merriam PA, Rosal MC, et al Seasonal Variation in Household, Occupational, and Leisure Time Physical Activity: Longitudinal Analyses from the Seasonal Variation of Blood Cholesterol Study. Am J Epidemiol. 2001;153: 172–183. 10.1093/aje/153.2.172 11159163

[22] Jones GR, Brandon C, Gill DP. Physical activity levels of community-dwelling older adults are influenced by winter weather variables. Arch Gerontol Geriatr. 2017;71: 28–33. 10.1016/j.archger.2017.02.012 28258987

[23] Quante M, Wang R, Weng J, Kaplan ER, Rueschman M, Taveras EM, et al Seasonal and weather variation of sleep and physical activity in 12-14-year-old children. Behav Sleep Med. 2017; 1–13. 10.1080/15402002.2017.1376206 PMC6214796 28922020

[24] Badland HM, Christian H, Giles-Corti B, Knuiman M. Seasonality in physical activity: should this be a concern in all settings? Health Place. 2011;17: 1084–1089. 10.1016/j.healthplace.2011.06.003 21742540

[25] Bélanger M, Gray-Donald K, O’Loughlin J, Paradis G, Hanley J. Influence of weather conditions and season on physical activity in adolescents. Ann Epidemiol. 2009;19: 180–186. 10.1016/j.annepidem.2008.12.008 19217000

[26] Duncan JS, Hopkins WG, Schofield G, Duncan EK. Effects of weather on pedometer-determined physical activity in children. Med Sci Sports Exerc. 2008;40: 1432–1438. 10.1249/MSS.0b013e31816e2b28 18614949

[27] Goodman A, Paskins J, Mackett R. Day Length and Weather Effects on Children’s Physical Activity and Participation in Play, Sports, and Active Travel. Journal of Physical Activity and Health. 2012 pp. 1105–1116. 10.1123/jpah.9.8.1105 22826506 PMC3584676

[28] Edwards NM, Myer GD, Kalkwarf HJ, Woo JG, Khoury PR, Hewett TE, et al Outdoor Temperature, Precipitation, and Wind Speed Affect Physical Activity Levels in Children: A Longitudinal Cohort Study. J Phys Act Health. 2015;12: 1074–1081. 10.1123/jpah.2014-0125 25423667 PMC4442747

[29] Yildirim M, Schoeni A, Singh AS, Altenburg TM, Brug J, De Bourdeaudhuij I, et al Daily variations in weather and the relationship with physical activity and sedentary time in European 10- to 12-year-olds: The ENERGY-Project. J Phys Act Health. 2014;11: 419–425. 10.1123/jpah.2012-0102 23363611

[30] Bunker A, Wildenhain J, Vandenbergh A, Henschke N, Rocklöv J, Hajat S, et al Effects of Air Temperature on Climate-Sensitive Mortality and Morbidity Outcomes in the Elderly; a Systematic Review and Meta-analysis of Epidemiological Evidence. EBioMedicine. 2016;6: 258–268. 10.1016/j.ebiom.2016.02.034 27211569 PMC4856745

[31] Song X, Wang S, Hu Y, Yue M, Zhang T, Liu Y, et al Impact of ambient temperature on morbidity and mortality: An overview of reviews. Sci Total Environ. 2017;586: 241–254. 10.1016/j.scitotenv.2017.01.212 28187945

[32] Gutiérrez-Avila I, Arfer KB, Wong S, Rush J, Kloog I, Just AC. A spatiotemporal reconstruction of daily ambient temperature using satellite data in the Megalopolis of Central Mexico from 2003–2018. 2019 [cited 28 Feb 2020]. 10.5281/zenodo.3362524 PMC8251982 34248276

[33] Bivand R, Lewin-Koh N. Package “maptools.” [cited 2 Oct 2019]. Available: https://cran.r-project.org/web/packages/maptools/maptools.pdf

[34] Fairclough SJ, Noonan R, Rowlands AV, Van Hees V, Knowles Z, Boddy LM. Wear Compliance and Activity in Children Wearing Wrist- and Hip-Mounted Accelerometers. Med Sci Sports Exerc. 2016;48: 245–253. 10.1249/MSS.0000000000000771 26375253

[35] Pereira JR, Cliff DP, Sousa-Sá E, Zhang Z, Santos R. Prevalence of objectively measured sedentary behavior in early years: Systematic review and meta-analysis. Scand J Med Sci Sports. 2019;29: 308–328. 10.1111/sms.13339 30456827

[36] Davison KK, Lawson CT. Do attributes in the physical environment influence children’s physical activity? A review of the literature. Int J Behav Nutr Phys Act. 2006;3: 19 10.1186/1479-5868-3-19 16872543 PMC1557665

[37] Evenson KR, Catellier DJ, Gill K, Ondrak KS, McMurray RG. Calibration of two objective measures of physical activity for children. J Sports Sci. 2008;26: 1557–1565. 10.1080/02640410802334196 18949660

[38] Song J, Cox MG. Package “acc.” [cited 2 Oct 2019]. Available: https://cran.r-project.org/web/packages/acc/acc.pdf

[39] Choi L, Liu Z, Matthews CE, Buchowski MS. Validation of accelerometer wear and nonwear time classification algorithm. Med Sci Sports Exerc. 2011;43: 357–364. 10.1249/MSS.0b013e3181ed61a3 20581716 PMC3184184

[40] Cain KL, Sallis JF, Conway TL, Van Dyck D, Calhoon L. Using Accelerometers in Youth Physical Activity Studies: A Review of Methods. J Phys Act Health. 2013;10: 437–450. 10.1123/jpah.10.3.437 23620392 PMC6331211

[41] Banda JA, Haydel KF, Davila T, Desai M, Bryson S, Haskell WL, et al Effects of Varying Epoch Lengths, Wear Time Algorithms, and Activity Cut-Points on Estimates of Child Sedentary Behavior and Physical Activity from Accelerometer Data. PLoS One. 2016;11: e0150534 10.1371/journal.pone.0150534 26938240 PMC4777377

[42] Renzetti S, Just AC, Burris HH, Oken E, Amarasiriwardena C, Svensson K, et al The association of lead exposure during pregnancy and childhood anthropometry in the Mexican PROGRESS cohort. Environ Res. 2017;152: 226–232. 10.1016/j.envres.2016.10.014 27810680 PMC5135667

[43] Burris HH, Baccarelli AA, Byun H-M, Cantoral A, Just AC, Pantic I, et al Offspring DNA methylation of the aryl-hydrocarbon receptor repressor gene is associated with maternal BMI, gestational age, and birth weight. Epigenetics. 2015;10: 913–921. 10.1080/15592294.2015.1078963 26252179 PMC4844209

[44] Chatterjee A, Thompson JW, Svensson K, Tamayo y Ortiz M, Wright R, Wright R, et al Maternal antenatal stress has little impact on child sleep: results from a prebirth cohort in Mexico City. Sleep Health. 2018;4: 397–404. 10.1016/j.sleh.2018.07.013 30241653 PMC6152833

[45] Johansson E, Larisch L-M, Marcus C, Hagströmer M. Calibration and Validation of a Wrist- and Hip-Worn Actigraph Accelerometer in 4-Year-Old Children. PLoS One. 2016;11: e0162436 10.1371/journal.pone.0162436 27617962 PMC5019366

[46] McClain JJ, Abraham TL, Brusseau TA Jr, Tudor-Locke C. Epoch length and accelerometer outputs in children: comparison to direct observation. Med Sci Sports Exerc. 2008;40: 2080–2087. 10.1249/MSS.0b013e3181824d98 18981941

[47] Yue Xu S, Nelson S, Kerr J, Godbole S, Patterson R, Merchant G, et al Statistical approaches to account for missing values in accelerometer data: Applications to modeling physical activity. Stat Methods Med Res. 2018;27: 1168–1186. 10.1177/0962280216657119 27405327

[48] WHO Child Growth Standards. 2006 [cited 2 Oct 2019]. Available: https://www.who.int/childgrowth/standards/Technical_report.pdf

[49] Sadeh A, Sharkey M, Carskadon MA. Activity-Based Sleep-Wake Identification: An Empirical Test of Methodological Issues. Sleep. 1994;17: 201–207. 10.1093/sleep/17.3.201 7939118

[50] Wood SN. Generalized Additive Models: An Introduction with R, Second Edition. CRC Press; 2017.

[51] Wood S. Package “mgcv.” [cited 2 Oct 2019]. Available: https://cran.r-project.org/web/packages/mgcv/mgcv.pdf

[52] Carson V, Hunter S, Kuzik N, Gray CE, Poitras VJ, Chaput J-P, et al Systematic review of sedentary behaviour and health indicators in school-aged children and youth: an update. Appl Physiol Nutr Metab. 2016;41: S240–65. 10.1139/apnm-2015-0630 27306432

[53] Husu P, Vähä-Ypyä H, Vasankari T. Objectively measured sedentary behavior and physical activity of Finnish 7- to 14-year-old children- associations with perceived health status: a cross-sectional study. BMC Public Health. 2016;16: 338 10.1186/s12889-016-3006-0 27083559 PMC4833900

[54] Pereira JR, Zhang Z, Sousa-Sá E, Santos R, Cliff DP. Correlates of sedentary time in young children: A systematic review. EJSS. 2020; 1–13. 10.1080/17461391.2020.1741689 32154761

[55] Song C, Gong W, Ding C, Yuan F, Zhang Y, Feng G, et al Physical activity and sedentary behavior among Chinese children aged 6–17 years: a cross-sectional analysis of 2010–2012 China National Nutrition and health survey. BMC Public Health. 2019 10.1186/s12889-019-7259-2 31296189 PMC6624983

[56] Ord K, Mitchell R, Pearce J. Is level of neighbourhood green space associated with physical activity in green space? Int J Behav Nutr Phys Act. 2013;10: 127 10.1186/1479-5868-10-127 24219824 PMC3833712

[57] U.S. Department of Health and Human Services. Physical Activity Guidelines for Americans, 2nd Edition. 2018 [cited 28 May 2020]. Available: https://health.gov/sites/default/files/2019-09/Physical_Activity_Guidelines_2nd_edition.pdf

[58] Dadvand P, Villanueva CM, Font-Ribera L, Martinez D, Basagaña X, Belmonte J, et al Risks and benefits of green spaces for children: a cross-sectional study of associations with sedentary behavior, obesity, asthma, and allergy. Environ Health Perspect. 2014;122: 1329–1335. 10.1289/ehp.1308038 25157960 PMC4256701

[59] Edwards N, Hooper P, Knuiman M, Foster S, Giles-Corti B. Associations between park features and adolescent park use for physical activity. Int J Behav Nutr Phys Act. 2015;12: 21 10.1186/s12966-015-0178-4 25879200 PMC4341879

[60] Dadvand P, Bartoll X, Basagaña X, Dalmau-Bueno A, Martinez D, Ambros A, et al Green spaces and General Health: Roles of mental health status, social support, and physical activity. Environ Int. 2016;91: 161–167. 10.1016/j.envint.2016.02.029 26949869

[61] Coombes E, Jones AP, Hillsdon M. The relationship of physical activity and overweight to objectively measured green space accessibility and use. Soc Sci Med. 2010;70: 816–822. 10.1016/j.socscimed.2009.11.020 20060635 PMC3759315

[62] Witten K, Hiscock R, Pearce J, Blakely T. Neighbourhood access to open spaces and the physical activity of residents: a national study. Prev Med. 2008;47: 299–303. 10.1016/j.ypmed.2008.04.010 18533242

[63] Choi J, Lee M, Lee J-K, Kang D, Choi J-Y. Correlates associated with participation in physical activity among adults: a systematic review of reviews and update. BMC Public Health. 2017;17: 356 10.1186/s12889-017-4255-2 28438146 PMC5404309

[64] Jankowska MM, Schipperijn J, Kerr J. A framework for using GPS data in physical activity and sedentary behavior studies. Exerc Sport Sci Rev. 2015;43: 48–56. 10.1249/JES.0000000000000035 25390297 PMC4272622

[65] Jones RA, Hinkley T, Okely AD, Salmon J. Tracking physical activity and sedentary behavior in childhood: a systematic review. Am J Prev Med. 2013;44: 651–658. 10.1016/j.amepre.2013.03.001 23683983

