# Supplementary figures and images for: Associations between daily ambient temperature and sedentary time among children 4–6 years old in Mexico City
Source: PLoS One. 2020 Oct 30;15(10):e0241446. doi: 10.1371/journal.pone.0241446 (PMC7598506; doi:10.1371/journal.pone.0241446)

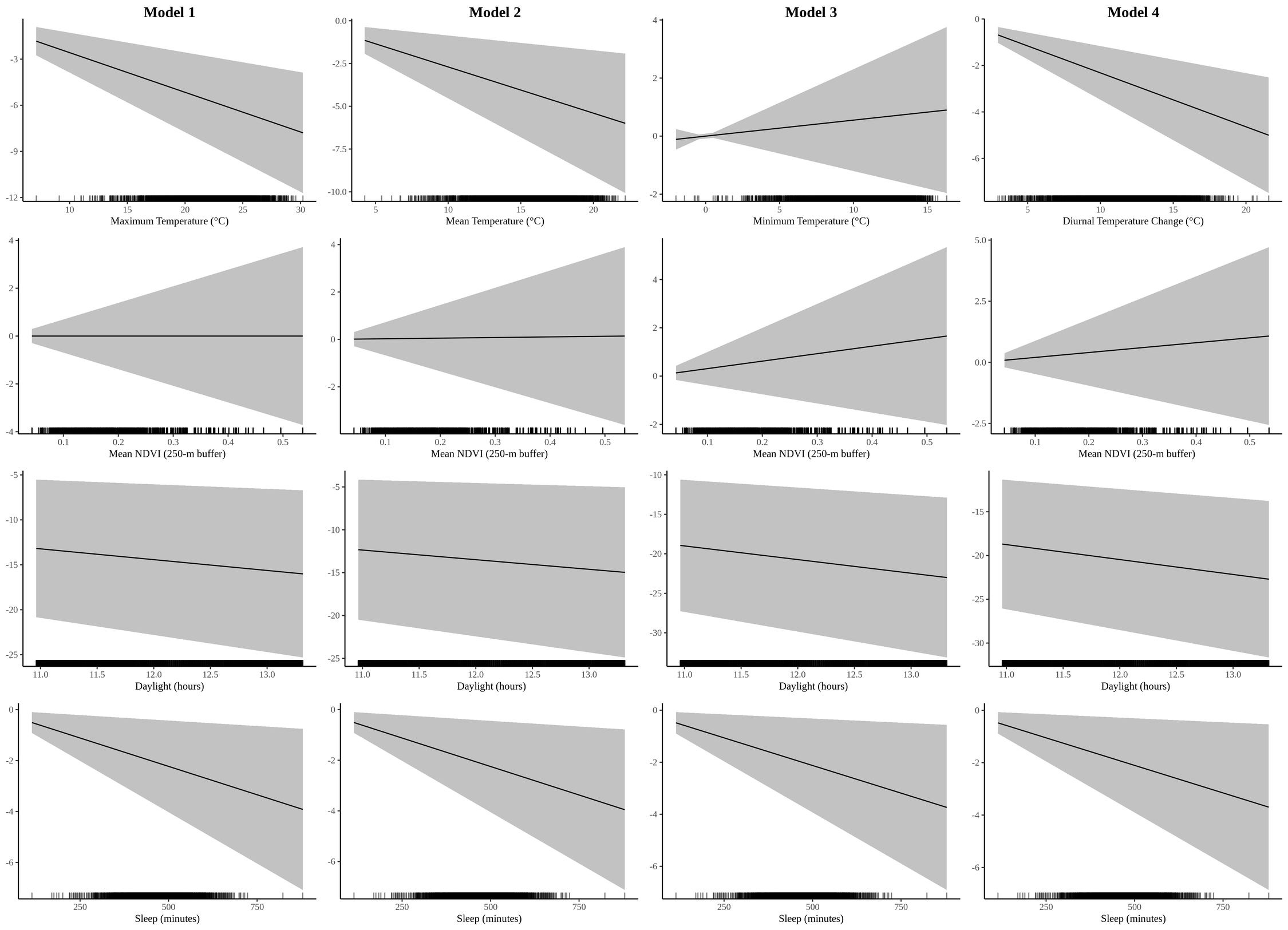

Supplement: S1 Fig — (TIF) [file pone.0241446.s001.tif]

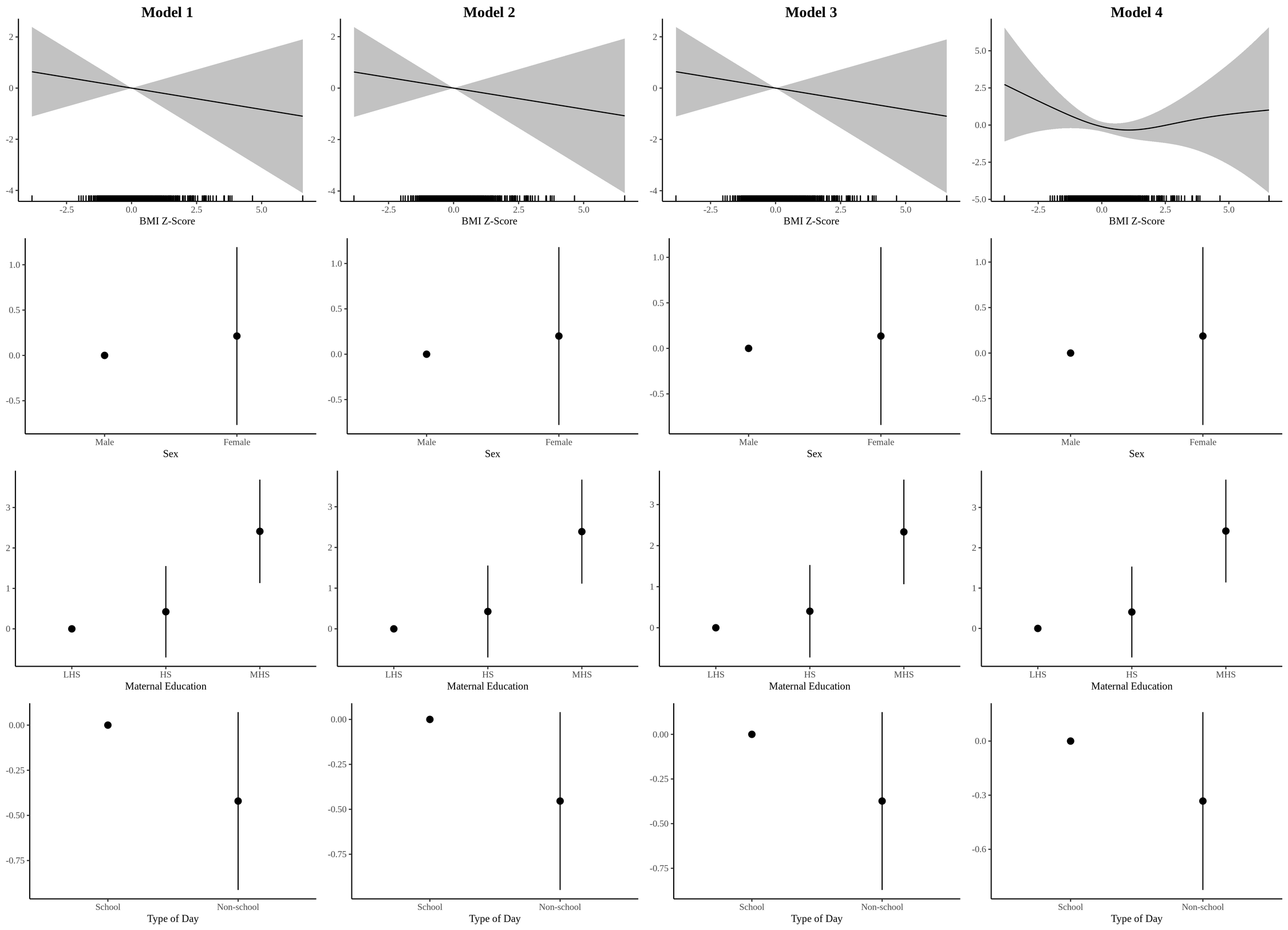

Supplement: S2 Fig — (TIF) [file pone.0241446.s002.tif]

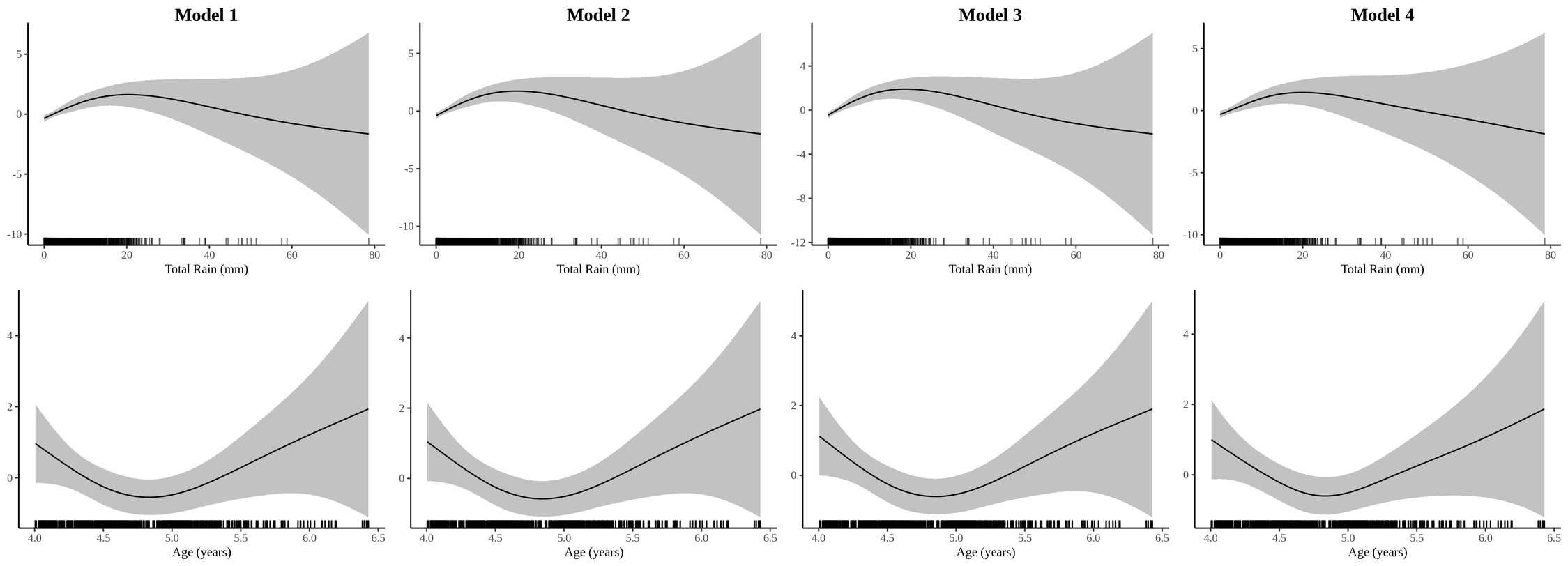

Supplement: S3 Fig — (TIF) [file pone.0241446.s003.tif]
