# Supplementary material for: Associations between daily ambient temperature and sedentary time among children 4–6 years old in Mexico City
Source: PLoS One. 2020 Oct 30;15(10):e0241446. doi: 10.1371/journal.pone.0241446 (PMC7598506; doi:10.1371/journal.pone.0241446)
Supplement: S1 Table — (DOCX) [file pone.0241446.s004.docx]

**S1 Table. Univariable GAMs results to assess non-linearity.**

| **Variable in Univariable GAM** | **Estimated Degrees of Freedom** | **AIC** |
| --- | --- | --- |
| s(Maximum Temperature) | 1.002 | 22333.6 |
| Maximum Temperature |  | 22333.53 |
| s(Mean Temperature) | 1.003 | 23675.44 |
| Mean Temperature |  | 23675.43 |
| s(Minimum Temperature) | 1.007 | 22361.45 |
| Minimum Temperature |  | 22361.12 |
| s(Diurnal Variation) | 2.127 | 22331.42 |
| Diurnal Variation |  | 22332.45 |
| s(NDVI) | 1.001 | 22360.1 |
| NDVI |  | 22360.01 |
| s(Daylight) | 1.003 | 22359.15 |
| Daylight |  | 22358.84 |
| s(Rain) | 2.347 | 22339.9 |
| Rain |  | 22350.14 |
| s(Sleep) | 1.001 | 22281.86 |
| Sleep |  | 22281.84 |
| s(Age) | 2.318 | 22359.83 |
| Age |  | 22360.01 |
| s(BMI Z-Score) | 1 | 22360.1 |
| BMI Z-Score |  | 22360.03 |
