# Supplementary material for: Associations between daily ambient temperature and sedentary time among children 4–6 years old in Mexico City
Source: PLoS One. 2020 Oct 30;15(10):e0241446. doi: 10.1371/journal.pone.0241446 (PMC7598506; doi:10.1371/journal.pone.0241446)
Supplement: S2 Table — (DOCX) [file pone.0241446.s005.docx]

**S2 Table. Univariable GAM results with daily percent sedentary time along y-axis.**

| **Variable** | **Estimate** | **95% CI** | **P-Value** |
| --- | --- | --- | --- |
| Maximum Temperature | -0.30 | -0.42, -0.18 | < 0.001 |
| (Intercept) | 62.6 | 59.9, 65.3 | < 0.001 |
| s(Participant) | 6.49 | 5.03, 5.82 | < 0.001 |
| Adjusted R^2^ | 0.41 |  |  |
| Mean Temperature | -0.33 | -0.49, -0.18 | < 0.001 |
| (Intercept) | 61.1 | 58.6, 63.6 | < 0.001 |
| s(Participant) | 5.40 | 5.02, 5.81 | < 0.001 |
| Adjusted R^2^ | 0.41 |  |  |
| Minimum Temperature | -0.01 | -0.16, 0.14 | 0.91 |
| (Intercept) | 56.1 | 54.5, 57.7 | < 0.001 |
| s(Participant) | 5.41 | 5.03, 5.82 | < 0.001 |
| Adjusted R^2^ | 0.41 |  |  |
| NDVI | 2.15 | -4.44, 8.74 | 0.522 |
| (Intercept) | 55.6 | 54.2, 56.9 | < 0.001 |
| s(Participant) | 5.41 | 5.03, 5.82 |  |
| Adjusted R^2^ | 0.41 |  |  |
| Daylight | -1.06 | -1.70, -0.41 | 0.001 |
| (Intercept) | 68.9 | 61.0, 76.9 | < 0.001 |
| s(Participant) | 5.35 | 4.97, 5.76 |  |
| Adjusted R^2^ | 0.41 |  |  |
| Sleep | -0.004 | -0.008, -0.0003 | 0.03 |
| (Intercept) | 57.9 | 56.1, 59.6 | < 0.001 |
| s(Participant) | 5.40 | 5.01, 5.81 | < 0.001 |
| Adjusted R^2^ | 0.41 |  |  |
| BMI Z-Score | -0.09 | -0.55, 0.37 | 0.70 |
| (Intercept) | 56.0 | 55.5, 56.5 | < 0.001 |
| s(Participant) | 5.41 | 5.03, 5.82 | < 0.001 |
| Adjusted R^2^ | 0.41 |  |  |
| s(Diurnal Variation) | 0.12 | 0.03, 0.53 | < 0.001 |
| (Intercept) | 56.0 | 55.5, 56.5 | < 0.001 |
| s(Participant) | 5.43 | 5.05, 5.85 | < 0.001 |
| Adjusted R^2^ | 0.41 |  |  |
| s(Rain) | 0.03 | 0.01, 0.11 | 0.001 |
| (Intercept) | 56.0 | 55.5, 56.5 | < 0.001 |
| s(Participant) | 5.44 | 5.06, 5.85 | < 0.001 |
| Adjusted R^2^ | 0.41 |  |  |
| s(Age) | 3.75 | 0.92, 15.23 | 0.06 |
| (Intercept) | 56.0 | 55.5, 56.5 | < 0.001 |
| s(Participant) | 5.36 | 4.98, 5.78 | < 0.001 |
| Adjusted R^2^ | 0.41 |  |  |
