# Supplementary material for: Associations between daily ambient temperature and sedentary time among children 4–6 years old in Mexico City
Source: PLoS One. 2020 Oct 30;15(10):e0241446. doi: 10.1371/journal.pone.0241446 (PMC7598506; doi:10.1371/journal.pone.0241446)
Supplement: S3 Table — (DOCX) [file pone.0241446.s006.docx]

**S3 Table. Additional GAM results adjusting for SIMAT mean temperature with daily percent sedentary time along y-axis.**

|  | ***SIMAT: Mean Temperature*** | | |
| --- | --- | --- | --- |
|  |  |  |  |
| **Variable** | **Estimate** | **95% CI** | **P-Value** |
| (Intercept) | 75.1 | 66.8, 83.4 | < 0.001 |
| Daily Temperature (°C) | -0.30 | -0.46, -0.12 | < 0.001 |
| Mean NDVI (250-m buffer) | 2.60 | -4.19, 9.39 | 0.45 |
| Daily Daylight (hours) | -1.08 | -1.82, -0.34 | 0.004 |
| Daily Sleep (minutes) | -0.004 | -0.008, -0.001 | 0.02 |
| BMI Z-Score | -0.17 | -0.62, 0.29 | 0.48 |
| Sex (Ref: Male) | 0.22 | -0.76, 1.20 | 0.66 |
| Maternal HS Education (Ref: LHS) | 0.39 | -0.74, 1.52 | 0.50 |
| Maternal More than HS Education (Ref: LHS) | 2.31 | 1.04, 3.59 | < 0.001 |
| Holiday/Weekend (Ref: School Day) | -0.45 | -0.94, 0.04 | 0.07 |
| s(Daily Total Rain) (mm) | 0.03 | 0.01, 0.11 | < 0.001 |
| s(Age) (years) | 3.40 | 0.83, 13.9 | 0.07 |
| s(Day of Year) | 8.9 x10^-5^ | 3.0 x10^-12^, 2.7 x10^3^ | 0.68 |
| s(Participant) | 5.21 | 4.84, 5.61 | < 0.001 |
| s(x,y) | 0.88 | 3.9 x10^-11^, 2.0 x10^10^ | 0.47 |
| Adjusted R^2^ | 0.42 | | |
|  |  |  |  |
| Abbreviation: LHS, Less than High School |  |  |  |
